# Identification and characterization of a novel *Fusobacterium nucleatum* adhesin involved in physical interaction and biofilm formation with *Streptococcus gordonii*

**DOI:** 10.1002/mbo3.444

**Published:** 2017-02-07

**Authors:** Bruno P. Lima, Wenyuan Shi, Renate Lux

**Affiliations:** ^1^ Division of Constitutive and Regenerative SciencesUniversity of California School of DentistryLos AngelesCAUSA; ^2^ Division of Oral Biology and MedicineUniversity of California School of DentistryLos AngelesCAUSA; ^3^ Present address: Bruno P. Lima, Department of Diagnostic and Biological SciencesSchool of DentistryUniversity of MinnesotaMinneapolisMNUSA

**Keywords:** Adhesin, *Fusobacterium nucleatum*, interspecies interaction, oral biofilm, *Streptococcus gordonii*

## Abstract

To successfully colonize the oral cavity, bacteria must directly or indirectly adhere to available oral surfaces. *Fusobacterium nucleatum* plays an important role in oral biofilm community development due to its broad adherence abilities, serving as a bridge between members of the oral biofilm that cannot directly bind to each other. In our efforts to characterize the molecular mechanisms utilized by *F. nucleatum* to physically bind to key members of the oral community, we investigated the involvement of *F. nucleatum* outer membrane proteins in its ability to bind to the pioneer biofilm colonizer, *Streptococcus gordonii*. Here, we present evidence that in addition to the previously characterized fusobacterial adhesin RadD, the interaction between *F. nucleatum* ATCC 23726 and *S. gordonii* V288 involves a second outer membrane protein, which we named *c*oaggregation *m*ediating *p*rotein *A* (CmpA). We also characterized the role of CmpA in dual‐species biofilm formation with *S. gordonii* V288, evaluated growth‐phase‐dependent as well as biofilm expression profiles of *radD* and *cmpA*, and confirmed an important role for CmpA, especially under biofilm growth conditions. Our findings underscore the complex set of specific interactions involved in physical binding and thus community integration of interacting bacterial species. This complex set of interactions could have critical implications for the formation and maturation of the oral biofilms in vivo, and could provide clues to the mechanism behind the distribution of organisms inside the human oral cavity.

## 1 INTRODUCTION

The human oral cavity is home to hundreds of bacterial species (Aas, Paster, Stokes, Olsen, & Dewhirst, 2005; Chen et al., 2010; Dewhirst et al., 2010; Kuramitsu, He, Lux, Anderson, & Shi, 2007; Paster et al., 2001). These organisms have coevolved and form a complex network of physical and metabolic interactions with their neighbors, as well as the human host. These connections promote the development of a dynamic and well‐organized multispecies microbial community, also known as the oral biofilm or dental plaque. Although a core set of organisms can be found in the oral cavity of most individuals, a variable microbiome exists in response to unique individual determinants (Paster, Olsen, Aas, & Dewhirst, 2006; Turnbaugh et al., 2007; Zaura, Keijser, Huse, & Crielaard, 2009). Depending on the combination of species present in the oral cavity, these communities can cause a multitude of oral and systemic diseases including dental caries, periodontal disease, and many others (Zarco, Vess, & Ginsburg, 2012).

To form these multispecies communities, oral bacteria must first, directly or indirectly, attach to a surface in the oral cavity. Oral streptococci are among the first species to attach to the surface of the teeth and they comprise the majority of the early colonizers (Avila, Ojcius, & Yilmaz, 2009; Diaz et al., 2006; Dige, Nilsson, Kilian, & Nyvad, 2007; Dige, Nyengaard, Kilian, & Nyvad, 2009; Nyvad & Kilian, 1987, 1990). Once established, the early colonizers alter their microenvironment and serve as anchors for subsequent colonizers of the dental plaque. The Gram‐negative bacterium *Fusobacterium nucleatum* is considered an important species in the development and maturation process of dental plaque (Jorth et al., 2014; Kolenbrander & London, 1993) as it contributes to important structural and metabolic changes. Structurally, *F. nucleatum* binds to numerous species in the oral cavity, serving as a bridge between early‐ and late‐colonizing species (Guo, He, & Shi, 2014; Kolenbrander, Andersen, & Moore, 1989; Kolenbrander, Parrish, Andersen, & Greenberg, 1995). Metabolically, *F. nucleatum* is a key contributor to butyrate production (Jorth et al., 2014), which has been linked to the development of periodontal disease (Niederman, Buyle‐Bodin, Lu, Robinson, & Naleway, 1997).

Earlier studies have focused on the identification and characterization of molecular components required for the direct cell‐to‐cell interaction among members of the oral community. Of special interest to our laboratory is the characterization of the interactions between *F. nucleatum* and other members of the dental plaque community. To date, only two *F. nucleatum* adhesins have been characterized for their role in interspecies interaction: Fap2 and RadD. Fap2 is a galactose‐inhibitable adhesin, which has been implicated in the interaction between *F. nucleatum* and the periodontal pathogen *Porphyromonas gingivalis* (Coppenhagen‐Glazer et al., 2015). RadD is an arginine‐inhibitable adhesin required for the interaction between *F. nucleatum* and multiple Gram‐positive members of the dental plaque, including the early colonizers *Actinomyces naeslundii*,* Streptococcus sanguinis*,* Streptococcus oralis*, and *Streptococcus gordonii* (Kaplan, Lux, Haake, & Shi, 2009).

In the work described here, we report the identification of a previously uncharacterized adhesin, which we named *c*oaggregation *m*ediating *p*rotein *A* (CmpA), involved in the interaction between *F. nucleatum* ATCC strain 23726 and *S. gordonii* V288. Along with RadD, CmpA plays an important role in the ability of *F. nucleatum* to coaggregate and form dual‐species biofilms with *S. gordonii* V288.

## 2 MATERIALS AND METHODS

### 2.1 Bacteria and culture conditions

All bacterial strains and plasmids used in this study are listed in Table 1. Unless otherwise stated, *F. nucleatum* strains were grown in Columbia broth or on Columbia agar plates (BD Difco, Detroit, MI) supplemented with 5% defibrinated sheep blood (Colorado Serum Company, Denver, CO) under anaerobic conditions (5% H_2_, 5% CO_2_, 90% N_2_) at 37°C. When necessary, Thiamphenicol and Clindamycin (MP Biomedicals, Irvine, CA) at 5 μg/ml and 0.2 μg/ml, respectively, were added to the media. For *P. gingivalis* growth, Columbia broth was supplemented with hemin and menadione at 5 μg/ml and 1 μg/ml, respectively. Columbia agar plates were supplemented with 5% defibrinated sheep blood. *S. sanguinis* and *S. gordonii* were grown in Todd Hewitt (TH) broth or agar plates (BD Difco, Detroit MI) at 37°C under anaerobic conditions. *Streptococcus gordonii* selection was carried out with 5 μg/ml erythromycin added to the media. *Escherichia coli* was grown aerobically at 37°C in Luria–Bertani (LB) broth or agar plates (BD Difco, Detroit, MI). *Escherichia coli* selection was carried out with 100 μg/ml erythromycin or ampicillin added to the media.

**Table 1:** Bacterial strains and plasmids used in this study

Species	Strains	Description	Source
*Escherichia coli*	DH10B™	F‐ *mcrA* Δ(*mrr‐hsd*RMS‐*mcr*BC) Φ80*lac*ZΔM15 Δ *lac*X74 *rec*A1* ara*D139 Δ(*araleu*)7697 *gal*U *gal*K *rps*L (StrR) *end*A1 *nup*G	Thermo Fisher
*Fusobacterium nucleatum*	ATCC 23726	ssp. *nucleatum* wild type	ATCC
	Δ*Fn0254*	ATCC 23726 *Fn0254*::pIP*0254*	Kaplan et al. (2009)
	Δ*Fn1526* (*radD)*	ATCC 23726 *Fn1526*::pIP*1526*	Kaplan et al. (2009)
	Δ*Fn1554* (*cmpA*)	ATCC 23726 Fn1554::pIP1554	Kaplan et al. (2009)
	Δ*Fn1893*	ATCC 23726 *Fn1893*::pIP*1983*	Kaplan et al. (2009)
	Δ*Fn2047*	ATCC 23726 *Fn2047*::pIP*2047*	Kaplan et al. (2009)
	*aim1*	ATCC 23726 *aim1*::pIP*aim1*	Kaplan et al. (2009)
	BL83	ATCC 23726 *radD::catP fn1554::Erm*	This study
*Streptococcus gordonii*	ATCC 10558	*S. gordonii* wild type	ATCC
	ATCC 51656	*S. gordonii* wild type	ATCC
	DL1	*S. gordonii* wild type	ATCC
	V288	*S. gordonii* wild type	ATCC
	BL98	V288 *attB::mCherry*	This study
*Streptococcus sanguinis*	ATCC 10556	WT *S. sanguinis*	ATCC
*Porphyromonas gingivalis*	4612	WT *P. gingivalis*	Lamont et al. (1995)

Plasmid	Description	Purpose	Source
pHS70	*F. nucleatum* suicide vector; *ermB*	Suicide plasmid	S. Haake (unpublished data)
pBPL9	pHS31 with *Fn1554_* inserted into *SnaBI* site	Gene inactivation plasmid	This study
pVA8912:mCherry	pVA8912 carrying mCherry under the control of *ldh* promoter.		Vickerman et al. (2015)

### 2.2 Coaggregation assay

Coaggregations were performed in coaggregation buffer (CAB) (150 mmol/L NaCl, 1 mmol/L Tris, 0.1 mmol/L CaCl_2_, 0.1 mmol/L MgCl_2_) as previously described with minor modifications (Kaplan et al., 2009). Overnight cultures of *S. gordonii* and *F. nucleatum* were diluted 10‐fold into fresh medium in the morning and grown until they reached OD_600_ 1.5 for *S. gordonii* and OD_600_ 2.0 for *F. nucleatum*. Bacterial cells were washed with CAB and resuspended, also with CAB. Equal numbers of bacterial cells were combined to a final concentration of 2 × 10^9^ cells ml^−1^ in a 1.5 ml microcentrifuge tube. To account for autoaggregation, the cells were also incubated in the absence of the binding partner. The tubes were vortexed for 10 s and incubated for 10 min at room temperature. After incubation, the bacterial mixtures were centrifuged at low speed (100*g*) for 1 min to pellet cellular aggregates, while leaving the nonaggregated bacteria in suspension. The supernatant was then removed without disturbing the pellet, and the optical density of the recovered supernatant was measured at 600 nm. For coaggregation inhibition assays, 50 mmol/L of l‐arginine was added to the reaction tube containing the different *F. nucleatum* strains and vortexed before addition of the partner strain. Relative coaggregation was determined by subtracting the turbidity of the recovered supernatant after coaggregation from the turbidity of the cell mixture before coaggregation and dividing the results by the turbidity before coaggregation.

### 2.3 Strain construction

#### 2.3.1 *Fusobacterium nucleatum* radD cmpA double mutant

An internal gene fragment from *cmpA* was amplified via PCR using *Taq* DNA polymerase and the primer pair 1554F’ 5′‐GAATGGCAGGATTTGCTTCA‐3′ and 1554R’ 5′‐TTGGTTAGTTCCCTTTGCGTA‐3′ (Kaplan et al., 2009). The amplicon was subcloned into pJET2.1 (New England Biolabs, Ipswich, MA) according to the manufacturer's protocol, prior to cloning into the *F. nucleatum* suicide vector pHS70 (see Table 1 for plasmid details). The resulting integration vector, named pBPL9, was transformed into the *F. nucleatum radD* mutant strain as described previously (Haake, Yoder, Attarian, & Podkaminer, 2000). Mutants were selected on Columbia agar plates containing 5% blood and 0.2 μg/ml clindamycin. Clindamycin resistance was confirmed by restreaking colonies onto Columbia blood plates containing 0.2 μg/ml clindamycin. Thiamphenicol resistance, and therefore radD mutation was confirmed by patching clindamycin‐resistant colonies onto Columbia blood plates containing 5 μg/ml thiamphenicol. Mutants were also confirmed by PCR analysis.

#### 2.3.2 mCherry^+^ S. gordonii

To construct the mCherry‐expressing *S. gordonii* strain BL98, we transformed plasmid pVA8912 (Vickerman, Mansfield, Zhu, Walters, & Banas, 2015) into the wild‐type *S. gordonii* V288 according to previously published protocol (Warren, Lund, Jones, & Hruby, 2007), utilizing competence‐stimulating peptide N‐DVRSNKIRLWWENIFFNKK (Pepmic, Suzhou, China). The mCherry encoding gene was inserted into *S. gordonii attB* site and is expressed under the control of the *ldh* promoter (for a full description of the construct, see Vickerman et al., 2015). The mCherry expression had no effect on coaggregation or dual‐species biofilm formation with *F. nucleatum* 23726.

### 2.4 Biofilm growth

One milliliter of SHI‐FSMS (50% SHI medium, Tian et al., 2010, 25% filtered saliva, 0.5% mannose, 0.5% sucrose; de Avila et al., 2015) containing 1 × 10^8^ *F. nucleatum* cells and 5 × 10^4^ *S. gordonii* cells diluted from overnight cultures were added to the wells of a 24‐well polystyrene culture plates (Thermo Fisher Scientific, Waltham, MA, USA) and incubated overnight under anaerobic conditions (5% H_2_, 5% CO_2_, 90% N_2_) at 37°C. After overnight growth, the planktonic cells were removed and the biofilm was washed three times with 500 μl of pre‐reduced, sterile phosphate‐buffered saline (PBS). The biofilm that remained attached to the wells was either processed for confocal laser scanning microscopy (CLSM) analysis or collected for DNA isolation.

### 2.5 Confocal laser scanning microscopy

Overnight biofilm samples were fluorescently labeled with the nucleic acid staining dye SYTO9 (Invitrogen, Carlsbad, CA) according to manufacturer's instructions and visualized using a Leica SPE I inverted CLSM (Leica, Wetzlar, Germany). SYTO9 fluorescence was measured using an excitation of 488 nm and emission at 530 nm. mCherry was expressed from the chromosome of *S. gordonii* and detected using an excitation of 543 nm and emission at 600 nm. Image analysis was carried out with the open‐source image processing software, FIJI (Schindelin et al., 2012).

### 2.6 Nucleic acid isolation

Genomic DNA was extracted from biofilms using MasterPure™ DNA Purification Kit (Epicenter^®^, Madison, WI, USA) according to manufacturer's instructions. The concentration of purified bacterial DNA was determined by Nanodrop 2000 (Thermo Scientific, Waltham, MA, USA).

Total RNA was extracted using PureLinK™ RNA Mini kit (Thermo Fisher Scientific, Waltham, MA, USA) according to manufacturer's instructions. Genomic DNA contamination was removed from total RNA using Turbo DNA‐free™ kit (Thermo Fisher Scientific, Waltham, MA, USA) according to manufacturer's instructions and confirmed by PCR using 16S rRNA primers Bac1 and Bac2 (Rupf, Merte, & Eschrich, 1999).

### 2.7 qPCR

To quantify the relative proportions of each species in the respective dual‐species biofilms, previously designed species‐specific primer pairs were used (Park, Shokeen, Haake, & Lux, 2016). For *F. nucleatum* ATCC 23726 and its mutant derivatives, a portion of the *Fusobacterium*‐specific *fomA* gene was amplified with Fn‐F (forward) 5′AGAGTTTGATCCTGGCTCAG3′ and Fn‐R (reverse) 5′GTCATCGTGCACACAGAATTGCTG3′ primers. For *S gordonii*, srtA‐F (forward) 5′TATTATGGTGCTGGTACGATGAAAGAGACTC3′ and srtA‐R (Reverse) 5′TATAGATTTTCATACCAGCCTTAGCACGATC3′ primers were chosen to amplify a portion of *S. gordonii srtA* gene. Primer pairs were tested for possible cross‐reactivity with the other species and for amplification efficiency. The efficiency range observed was between 90% and 100%. Real‐time qPCR was performed using an iCycler Thermal Cycler (Bio‐Rad, Hercules, CA) in a total volume of 20 μl containing 2 μl of 10× iQ SYBR^®^ Green Supermix (Bio‐Rad, Hercules, CA), 0.5 μmol/L each of forward and reverse primers, 7 μl of Millipore water, and 1 μl (10 ng) of template DNA. Amplification and detection were carried out in 96‐well optical plates (Thermo Fisher Scientific, Waltham, MA). Each PCR run was carried out with an initial incubation of 10 min at 95°C followed by 40 cycles of denaturing at 95°C for 15 s and annealing and elongation at 60°C for 1 min. After the 40 cycles of amplification, an additional denaturing step was performed at 95°C for 1 min followed by annealing and elongation at 60°C for 1 min. A melting curve analysis was completed after each run. In addition, gel electrophoresis was utilized during optimization step to determine size and number of amplicons. The DNA concentrations (ng ml^−1^) were calculated with standard curves obtained by 10‐fold serial dilutions of previously purified and quantified bacterial genomic DNA. Three independent qPCR runs were performed with three technical replicates for each sample to assess reproducibility and inter‐run variability.

### 2.8 Transcriptional analysis

To determine *radD* and *cmpA* expression pattern, 1 μg of total RNA was used for cDNA synthesis using SuperScript^®^ III First‐Strand (Thermo Fisher Scientific, Waltham, MA, USA) according to manufacturer's protocol. For qRT‐PCR, iQ SYBR^®^ Green Supermix (Bio‐Rad, Hercules, CA, USA) was used for fluorescence detection with the iCycler real‐time PCR system (Bio‐Rad), according to the manufacturer's instructions. *radD* cDNA was amplified using 5′‐GGATTTATCTTTGCTAATTGGGGAAATTATAG‐3′ forward and 5′‐ACTATTCCATATTCTCCATAATATTTCCCATTAGA‐3′ reverse primers (B. Shokeen, J. Park, and R. Lux unpublished data), and *cmpA* was amplified using 5′‐TTGGGATCAAGGAAAACATCAATTAGG‐3′ forward and 5′‐ATAATTCCTTTATTATCTCCCATATAAGCAATACC‐3′ reverse primers. Expression levels of *rpoB* were determined using 5′‐CAAAAACTCATTGAAAGACTTGATTTTGGA‐3′ forward and 5′‐GAATGCTAATTCAAATCCTTTTTCTTCCCT‐3′ reverse primers for normalization of the qRT‐PCR data (B. Shokeen, J. Park, and R. Lux unpublished data).

### 2.9 Statistical analysis

Student's *t*‐test was performed to determine statistical significance using Excel 2010 (Microsoft, Seattle, WA, USA).

## 3 RESULTS

### 3.1 Multiple *F. nucleatum* adhesins are involved in its coaggregation with *S. gordonii*

**Figure 1:**
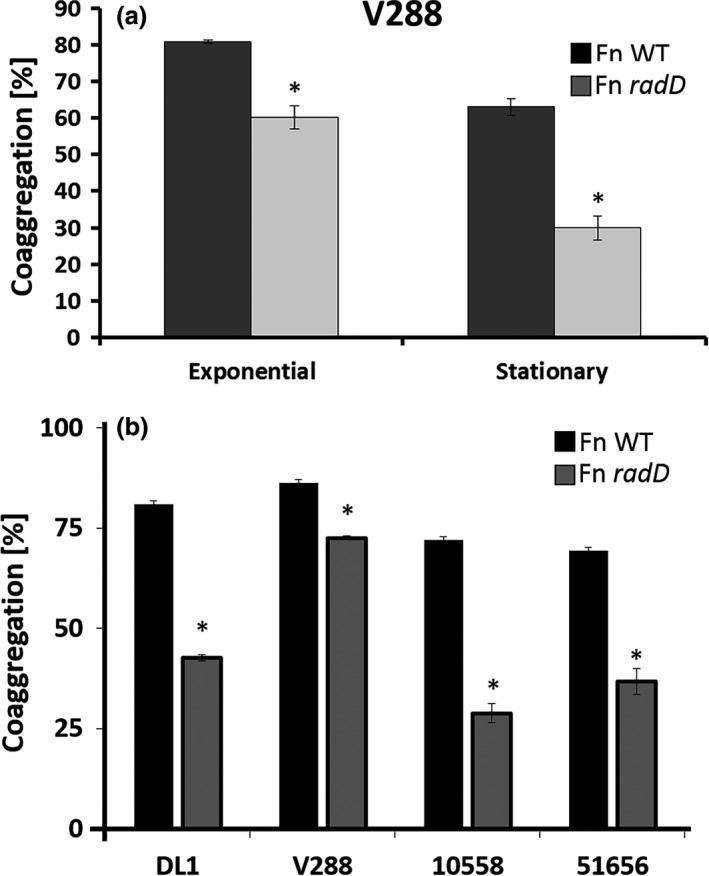
Quantitative coaggregation of (a) wild‐type (WT) *Streptococcus gordonii* strain V288 and the WT *Fusobacterium nucleatum* strain ATCC 23726, or the *radD* mutant derivative, at different phases of growth. (b) Quantitative coaggregation of WT *S. gordonii* strains (DL1, V288, ATCC 10558, and ATCC 51656) with WT *F. nucleatum* strain ATCC 23726, or the *radD* mutant derivative at exponential growth. Data represent means and standard deviation of percent coaggregation of at least three independent experiments. *p < .05 compared to wild‐type control

For detailed characterization of the physical interaction between *F. nucleatum* and *S. gordonii*, we collected *F. nucleatum* cells, ATCC strain 23726, that were in stationary phase, after overnight growth, and cells that were in late exponential phase to perform coaggregation experiments with midexponential phase *S. gordonii* V288 cells. We observed less coaggregation with stationary phase *F. nucleatum* cultures (63.03% ± 2.39) when compared to those in late exponential phase (80.85% ± 0.5) (Figure 1a). Surprisingly, the coaggregation defect usually observed for the *radD* mutant derivative of *F. nucleatum* was less evident at the late exponential phase, compared to *F. nucleatum* collected the stationary growth phase (Figure 1a). This phenotype was not evident among other commonly used *S. gordonii* strains (DL1, ATCC 10558, and ATCC 51656) (Figure 1b). Thus, *F. nucleatum* coaggregation with *S. gordonii* V288 might require an additional, and yet uncharacterized, surface adhesin that is likely expressed as cells enter the exponential phase of growth.

### 3.2 Identification of an additional adhesin involved in the interaction between *F. nucleatum* and *S. gordonii*

**Figure 2:**
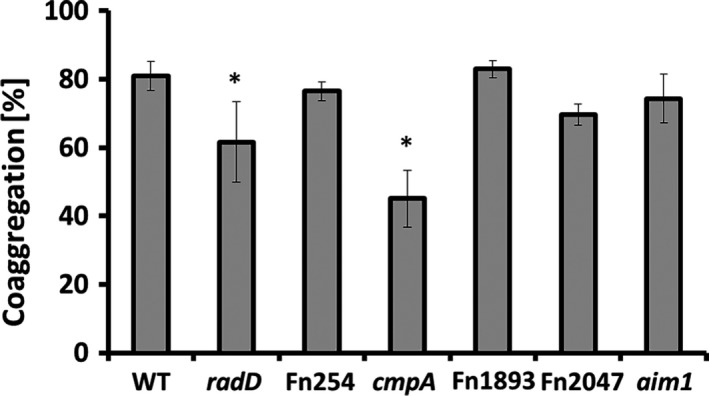
Quantitative coaggregation of wild‐type (WT) *Streptococcus gordonii* strain V288 with *Fusobacterium nucleatum* ATCC 23726WT strain and OMP mutant derivatives (*radD*, Fn254, *cmpA*, Fn1893, Fn2047, and *aim1*). Data represent means and standard deviation of percent coaggregation of at least three independent experiments. *p < .05 compared to wild‐type control

The genome of the sequenced *F. nucleatum* strain ATCC 25586 encodes at least eight large autotransporter‐like outer membrane proteins (OMPs): Fn0254, Fn0387, Fap2 (Fn1449), RadD (Fn1526), Fn1554, Fn1893, Fn2047, and Aim1 (Fn2058) (Kaplan et al., 2009), of which, six have been shown to bind arginine: Fn0254, RadD, Fn1554, Fn1893, Fn2047, and Aim1 (Kaplan et al., 2009). Since the interaction between *F. nucleatum* (ATCC 23726) and *S. gordonii*, including strain V288, is inhibited by the presence of 50 mmol/L arginine (Figure 4a of this manuscript; Kaplan et al., 2009), we hypothesized that the additional *F. nucleatum* adhesin involved in its interaction with *S. gordonii* was one of the arginine‐binding adhesins. Therefore, we screened the remaining five previously identified arginine‐binding adhesins (Fn0254, Fn1554, Fn1893, Fn2047, or Aim1) for their ability to coaggregate with *S. gordonii* V288. The Fn1554 mutant strain of *F. nucleatum* consistently coaggregated less with *S. gordonii* V288 than the wild‐type parent strain or any of the other mutants tested (Figure 2). Thus, we concluded that Fn1554 (now named CmpA for *c*oaggregation *m*ediating *p*rotein *A*) and RadD are important *F. nucleatum* coaggregation mediating proteins involved in its interaction with *S. gordonii* V288.

**Figure 3:**
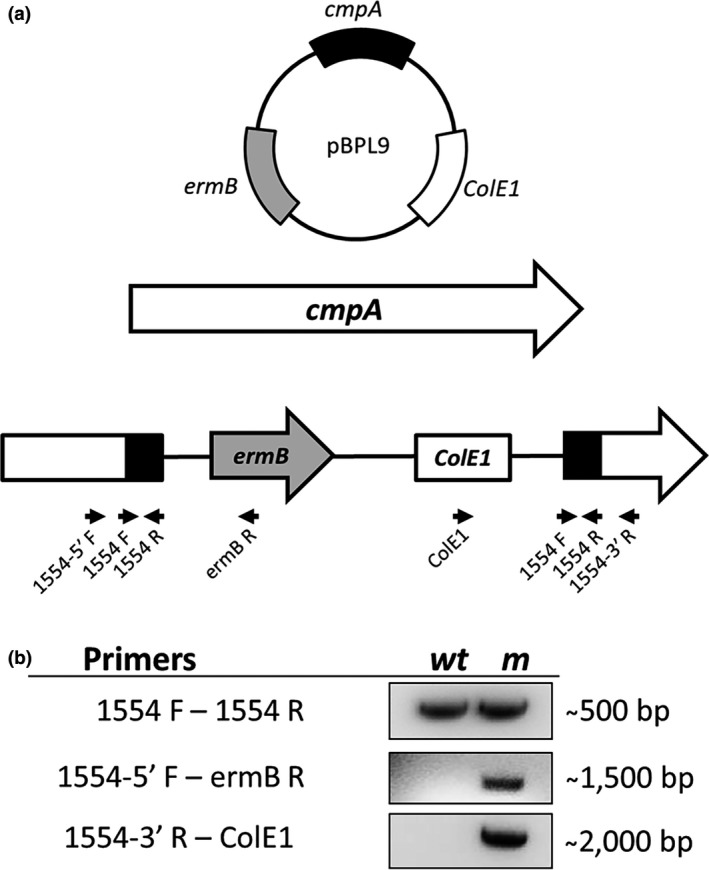
(a) *Fusobacterium nucleatum radD cmpA* double mutant (strain BL83) was constructed by insertion of the inactivation plasmid pBPL9 into *cmpA* (Fn1554) in a *radD::catP* mutant background. Black arrows indicate the location of primers used for mutant construction and analysis. (b) Confirmation of plasmid insertion into *cmpA* by PCR analysis of the *cmpA* mutant (m) with wild‐type (WT) as the control

**Figure 4:**
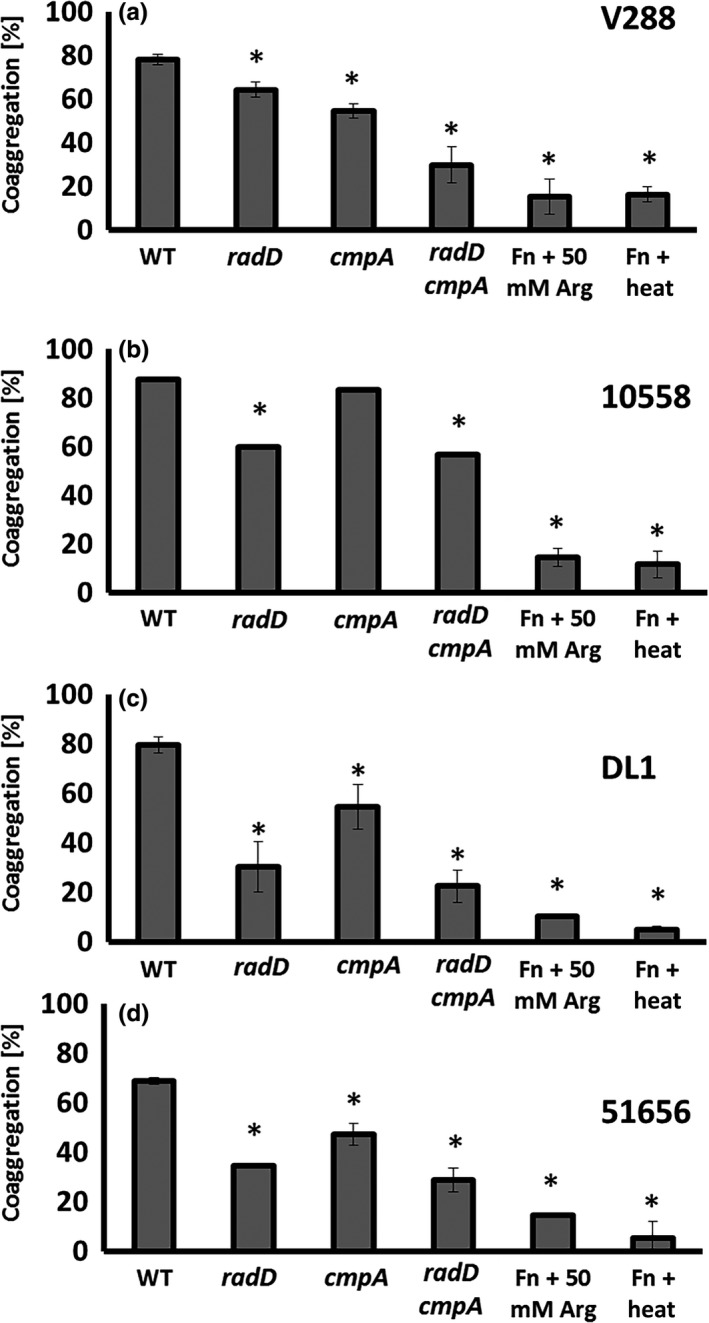
Quantitative coaggregation of wild‐type *Streptococcus gordonii* strain (a) V288, (b) ATCC 10558, (c) DL1, and (d) ATCC 51656 with *Fusobacterium nucleatum* strains: Wild‐type (WT) and the mutant derivatives: *radD*,* cmpA*, and *radD cmpA* double mutant. Data represent means and standard deviation of percent coaggregation of at least three independent experiments. *p < .05 compared to wild‐type control

The individual absence of the proteins encoded by *radD* and *cmpA* only resulted in a partial coaggregation defect with *S. gordonii*. For further characterization of these OMPs in binding to *S. gordonii* V288, we constructed a double *radD cmpA* mutant, by using previously established approaches (Haake et al., 2000; Kaplan et al., 2005, 2009) to inactivate the ORF encoding *cmpA* in the *radD* mutant background (Figure 3a and b). Consistent with the idea that *cmpA* and *radD* function independently from each and are the two major adhesins involved in the *F. nucleatum* 23726 interaction with *S. gordonii* V288, the double mutant had a stronger coaggregation defect than either single mutant, and the defect in coaggregation was similar to that observed when the coaggregation inhibitor arginine was added to the buffer (Figure 4a).

### 3.3 CmpA coaggregation involvement is *S. gordonii* strain dependent

In contrast to above findings with *S. gordonii* V288, we had previously failed to observe a CmpA involvement in the interaction between *F. nucleatum* 23726 and *S. gordonii* ATCC10588, which was used as a representative of this oral *Streptococcus* species in one of our earlier studies (Kaplan et al., 2009). Thus, we decided to test the possible involvement of CmpA in the coaggregation between *F. nucleatum* and other *S. gordonii* strains such as ATCC 51656 and DL110558 as well as the previously used ATCC 10588. We found that the interaction of *F. nucleatum* with *S. gordonii* ATCC 10558 does not seem to involve CmpA (Figure 4b), whereas strains DL1 and ATCC 51656 presented only a subtle defect in coaggregation (Figure 4c and d).

We also investigated if CmpA was involved in *F. nucleatum* interaction with the periodontal pathogen *P. gingivalis* (strain 4612) and with another streptococcal species closely related to *S. gordonii* and *S. sanguinis* (ATCC 10556). However, we did not observe any difference in coaggregation compared to the wild‐type strains (data not shown). Thus, to the extent that we have tested, CmpA seems to be largely involved in the specific interaction between *F. nucleatum* strain 23726 and *S. gordonii* V288.

### 3.4 CmpA is required for dual‐species biofilm formation with *S. gordonii*

**Figure 5:**
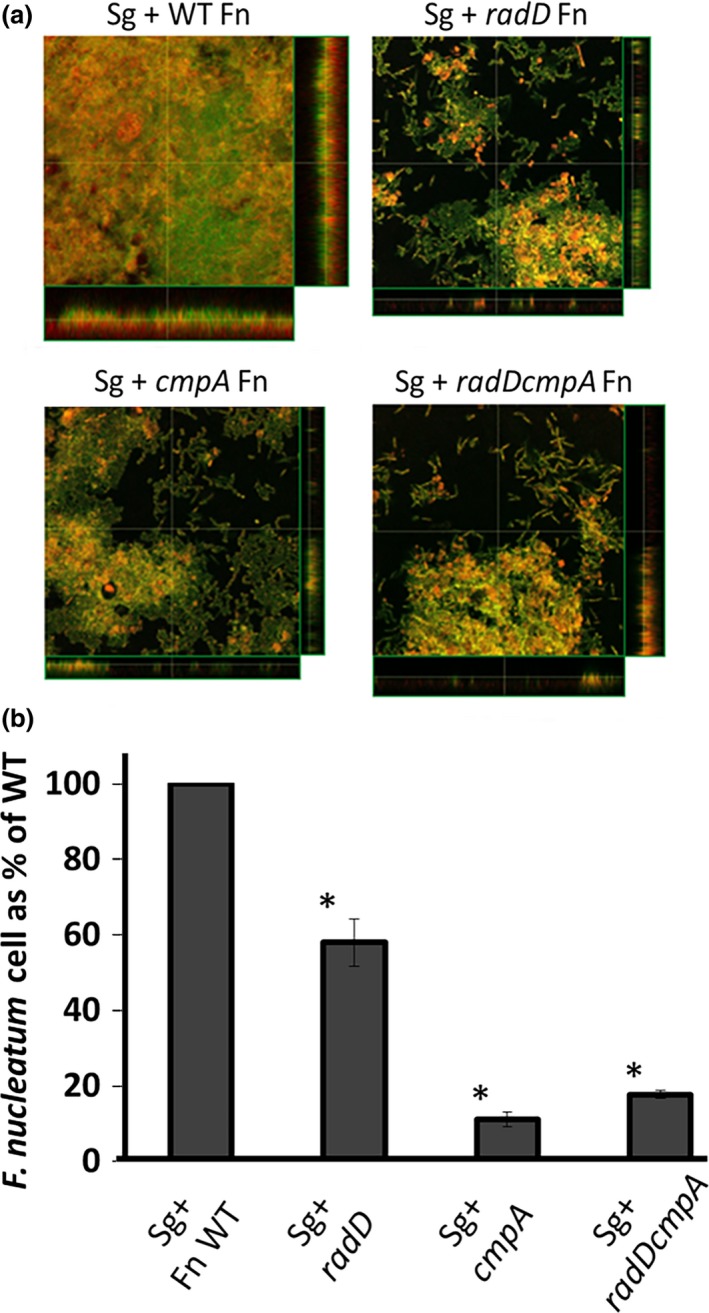
*Streptococcus gordonii* and *Fusobacterium nucleatum* dual‐species biofilm: (a) Confocal laser scanning microscopy of syto9‐stained dual‐species biofilm after overnight incubation. *S. gordonii* (Sg) cells constitutively express mCherry from their chromosome and appear orange/yellow on the images. Wild‐type (WT) *F. nucleatum* (Fn) and its mutant derivatives (*radD*,* cmpA*, and *radD cmpA* double mutant) are strained by syto9‐only and appear green on the images. (b) The presence of the Fn mutant strains in the Sg‐Fn dual‐species biofilm is displayed as the percentage of Fn cells normalized to the number of attached Sg cells/well, compared to that measured with WT Fn. Cellular ratios were determined by measuring DNA concentration by qPCR, targeting the *F. nucleatum* gene *fomA* and the *S. gordonii* gene *srtA*. At least three independent experiments were performed per strain combination. Each value represents means and standard deviation of at least three independent experiments. *p < .05 compared to wild‐type control

The interaction between streptococcal species and *F. nucleatum* is hypothesized to be an important step in oral biofilm development (Kolenbrander, 1989; Kolenbrander & London, 1993). Previous studies demonstrate that *F. nucleatum* requires *radD* to form a dual‐species biofilm with at least one of the oral streptococci species, *S. sanguinis* (Kaplan et al., 2009; Lancy, Dirienzo, Appelbaum, Rosan, & Holt, 1983). Since our data demonstrate that both RadD and CmpA are involved in the physical binding between *F. nucleatum* and *S. gordonii* V288, we investigated whether these adhesins were also involved in dual‐species biofilm formation. To differentiate between *S. gordonii* and *F. nucleatum*, we utilized an mCherry‐expressing *S. gordonii* V288 strain (BL98). While wild‐type *F. nucleatum* formed a uniform and thick biofilm layer on top of the mCherry‐expressing *S. gordonii* cells, the adhesin mutants, both single and double mutants, formed an irregular and thinner biofilm, as determined by confocal laser scanning microscopy (Figure 5a). Analysis of three randomly selected biofilm images revealed that the average height and maximum height of the biofilms were 38.21 μm (±11.48) and 241.33 μm (±23.67) for the WT *F. nucleatum*; 6.88 μm (±1.87) and 163.33 μm (±41.86) for the radD mutant; 8.47 μm (±0.19) and 171.66 μm (±48.41) for the cmpA mutant; and 4.48 μm (±1.04) and 71.66 μm (±34.93) for the double mutant.

qPCR analysis of the total DNA extracted from the dual‐species biofilm revealed a decrease in the ratio of *F. nucleatum* to *S. gordonii* cells, as measured by their respective relative DNA concentration, when *radD* or *cmpA* mutants were used, compared to wild‐type cells (Figure 5b). Similar results were observed for the *radD cmpA* double mutant (Figure 5b).

### 3.5 *radD* and *cmpA* expression pattern

**Figure 6:**
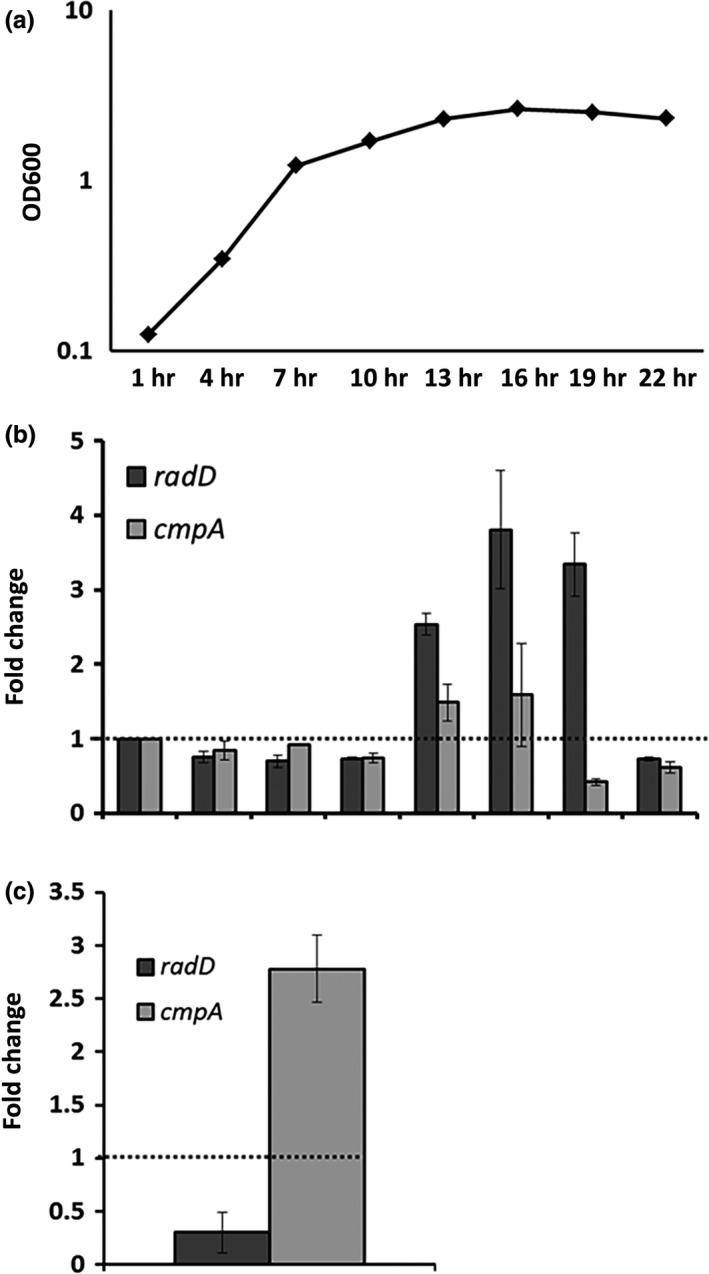
*radD* and *cmpA* expression: WT *Fusobacterium nucleatum* was grown in Columbia broth for 25 hr. Cell samples were collected every 3 hr and (a) OD600 was measured, as well as (b) *radD* and *cmpA* expression by qRT‐PCR. Gene expression was normalized to rpoB and compare to the first time point. The dashed line was added to aid in the comparison. (c) *radD* and *cmpA* expression were also measured in cells from Fn biofilm grown overnight in SHI‐FSMS. Gene expression was normalized to *rpoB* and compare to planktonic cells grown under the same conditions. Each value represents means and standard deviation of at least three independent experiments. To aid with visualization, the dashed line represents no change

To further characterize *radD* and *cmpA*, we measured their mRNA level in wild‐type *F. nucleatum* throughout planktonic growth in Columbia broth from the early exponential to the stationary phase as well as from a single time point from the overnight biofilm. Both *radD* and *cmpA* mRNA level increased as the cultures entered stationary phase, but quickly decreased in the following time points (Figure 6a and b) implicating a growth‐dependent regulation of adhesins expression. Under overnight biofilm conditions, *radD* expression was about threefold lower while *cmpA* expression was about threefold higher compared to the planktonic cells collected from the same wells (Figure 6c).

## 4 DISCUSSION

The organization of the oral microbial community is thought to involve a complex network of interactions, often mediated by surface adhesins. As part of our long‐term effort to characterize the physical interaction between *F. nucleatum* and other members of the oral microbial community, we investigated, at the molecular level, the interaction between *F. nucleatum* 23726 and one of its early‐colonizer partners, *S. gordonii*. Here, we provide evidence that the physical interaction between *F. nucleatum* 23726 and *S. gordonii* V288 is mediated by at least two arginine‐inhibitable adhesins, RadD and CmpA.

The involvement of CpmA in the interaction between *F. nucleatum* 23726 and *S. gordonii* was most relevant for strain V288 compared to other *S. gordonii* strains tested (DL1, ATCC 10558, and ATCC 51656) (Figure 4a–d). The difference in coaggregation phenotype between *S. gordonii* V288 and DL1 was surprising, since these two strains are derived from the same original isolate, *S. gordonii* Challis. This has made us wonder how easily *S. gordonii* could alter its adhesion properties. These data also explain why we failed to observe a CmpA involvement in our previously published screen, which used strain ATCC 10558 as the *S. gordonii* representative strain for identification of *F. nucleatum* adhesins (Kaplan et al., 2009). Most significantly, these data add an additional layer of complexity to the interaction between *F. nucleatum* and *S. gordonii*. Similar complexity seems to be present in the interaction between *F. nucleatum* and *P. gingivalis*; while Fap2 appears to be a major adhesin for the interaction of *F. nucleatum* with *P. gingivalis* (Coppenhagen‐Glazer et al., 2015; Park et al., 2016), RadD plays an additional role in binding to strain 4612 (Park et al., 2016). It is worth mentioning that neither RadD nor Fap2 is involved in the interaction between *F. nucleatum* and *P. gingivalis* strain ATCC 33277, implicating the existence of at least one more *F. nucleatum* adhesin involved in the *F. nucleatum*–*P. gingivalis* interaction (Park et al., 2016).

While it is widely accepted that the initial interaction between oral bacteria can be assessed in vitro by measuring the ability of their planktonic cells to coaggregate, in the oral cavity, the ability of individual cells or group of cells to integrate into a biofilm is crucial for their survival and maintenance in the oral cavity (Kolenbrander, Palmer, Periasamy, & Jakubovics, 2010). Cells that integrate into a biofilm undergo significant physiological changes compared to their planktonic counterparts. These changes include, but are not limited to (1) gene and protein expression patterns, (2) metabolic preferences, and (3) replication rates (Cook, Costerton, & Lamont, 1998; Resch, Rosenstein, Nerz, & Gotz, 2005). Our results demonstrate that during formation of dual‐species biofilm with *S. gordonii* V288 in vitro, both RadD and CmpA are key players (Figure 5a and b). Interestingly, while *cmpA* expression was increased under biofilm conditions, *radD* expression was decreased (Figure 6c), suggesting that these proteins are likely to be involved in different physiological processes under biofilm conditions. Perhaps, RadD could play a more important role in the initial binding, while CmpA could be more involved in the subsequent stages of biofilm development.

*Fusobacterium nucleatum* encodes at least eight autotransporter‐like OMPs with molecular weights greater than 200 kDa. RadD, Fap2, and Aim1 have been previously characterized as interspecies adhesins (Coppenhagen‐Glazer et al., 2015; Kaplan et al., 2009) and apoptosis‐inducing proteins (Kaplan et al., 2005). The involvement of CmpA in interspecies interaction leaves five OMPs to be characterized. These autotransporter‐like OMPs possess very similar characteristics and are predicted to contain a core β‐barrel structure (Kaplan et al., 2009). In other bacteria, this structure possesses multiple activities that function in adherence and biofilm formation (Charbonneau & Mourez, 2007; Korotkova et al., 2006; Laarmann, Cutter, Juehne, Barenkamp, & ST Geme, 2002). The wide array of adherence properties found in *F. nucleatum* strains could be mediated by a combination of OMPs with varying degrees of affinity for different species and/or strains of bacteria present in the oral cavity.

In summary, the data presented here support the existence of a potentially complex interaction network between *F. nucleatum* and *S. gordonii*, which seems to be mediated by varying degrees of preferences for different *F. nucleatum* adhesins. This variation in adhesin preference could have a profound impact on community composition and species distribution in the oral microbiome, especially if such phenotype is also observed in the interaction among other members of the oral microbial community.

## CONFLICT OF INTEREST

None declared.
